# Delivery of the reduced form of vitamin K_2(20)_ to NIH/3T3 cells partially protects against rotenone induced cell death

**DOI:** 10.1038/s41598-022-24456-3

**Published:** 2022-11-18

**Authors:** Erina Toki, Shotaro Goto, Shuichi Setoguchi, Kazuki Terada, Daisuke Watase, Hirofumi Yamakawa, Ayano Yamada, Mitsuhisa Koga, Kaori Kubota, Katsunori Iwasaki, Yoshiharu Karube, Kazuhisa Matsunaga, Jiro Takata

**Affiliations:** 1 Faculty of Pharmaceutical Sciences, Fukuoka University, Fukuoka, 814-0180 Japan; 2 Faculty of Pharmaceutical Sciences, Himeji Dokkyo University, Himeji, 670-8524 Japan; 3 Radioisotope Center, Fukuoka University, Fukuoka, 814-0180 Japan

**Keywords:** Mitochondria, Drug delivery

## Abstract

Mitochondria generate energy through the action of the electron transport chain (ETC) and ATP synthase. Mitochondrial malfunction can lead to various disorders, including neurodegenerative diseases. Several reports have shown that menaquinone-4 (MK-4, vitamin K_2(20)_), a safe drug for osteoporosis, may improve mitochondrial function. Here, we hypothesized that the efficient delivery of menahydroquinone-4 (MKH), an active form of MK-4, could exert a supporting effect. We verified the effects of MKH delivery on mitochondrial dysfunction by using MK-4 and MKH ester derivatives in NIH/3T3 mouse fibroblast cells treated with mitochondrial inhibitors. MK-4 and MKH derivatives suppressed cell death, the decline in mitochondrial membrane potential (MMP), excessive reactive oxygen species (ROS) production, and a decrease in intrinsic coenzyme Q_9_ (CoQ_9_) induced by rotenone (ROT, complex I inhibitor). MK-4 and MKH derivatives delivered MKH to NIH/3T3 cells, acting as an effective MKH prodrug, proving that the delivered MKH may reflect the mitigation effects on ROT-induced mitochondrial dysfunction. MKH prodrugs are also effective against 3-nitropropionic acid (3-NP, complex II inhibitor) and carbonyl cyanide-m-chlorophenylhydrazone (CCCP, uncoupler)-induced cell death. In conclusion, MKH delivery may mitigate mitochondrial dysfunction by maintaining MMP, ROS, and CoQ_9_, indicating that MKH prodrugs may be good candidates for treating mitochondrial disorders.

## Introduction

Mitochondria are integral for normal cell functioning as they generate the ATP required to maintain vital cell function in the respiratory chain behaving as “powerhouses of the cell.” Mitochondrial quality control and dysfunction are also implicated in the production of reactive oxygen species (ROS), regulation of cell death, and etiology of neurological disorders such as Huntington's disease, Parkinson's disease, and Alzheimer's disease^1–3^. Therefore, focusing on mitochondrial function is necessary to elucidate the etiology of diseases for which there are still no effective treatment strategies and advance effective drug development.

**Figure 1:**
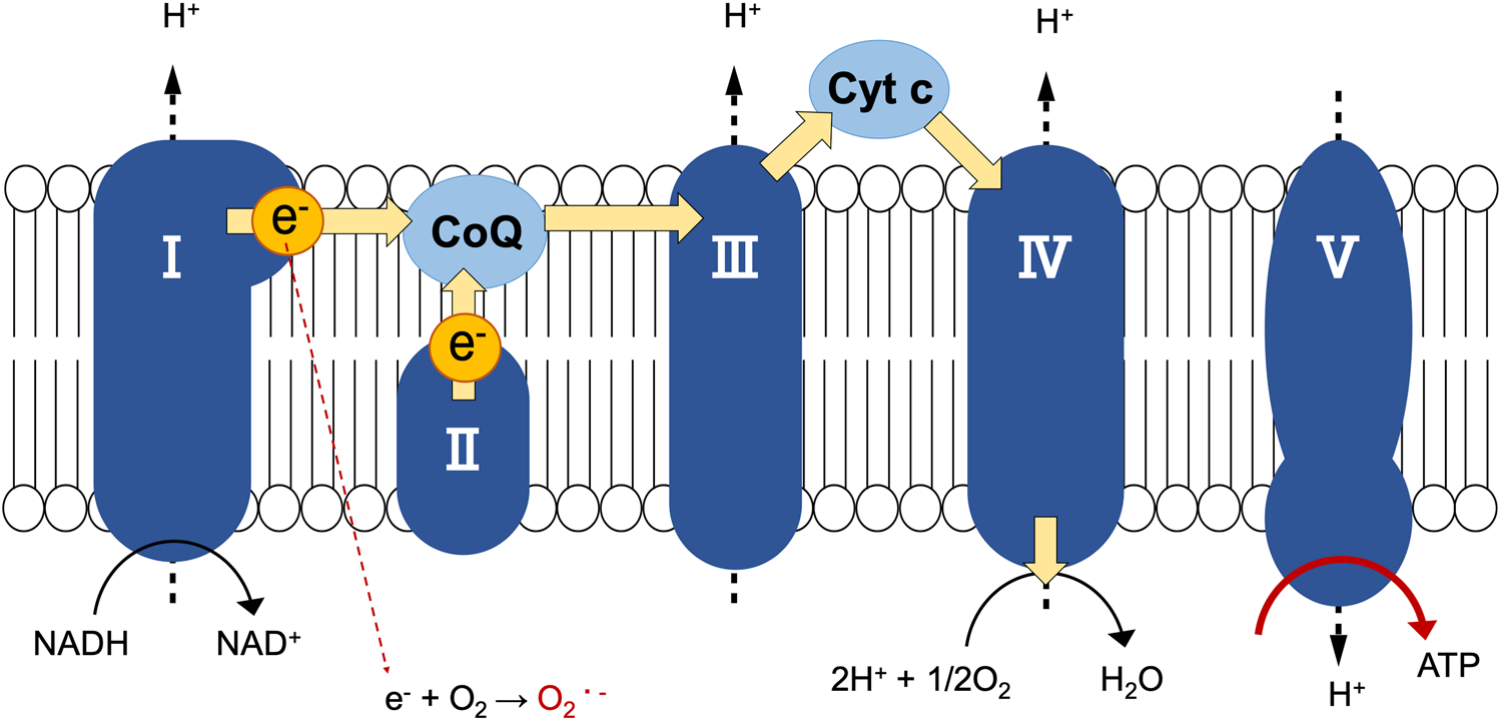
Mitochondrial respiratory chain complexes. Respiratory chain complexes I–IV generate a proton gradient on the mitochondrial inner membrane that facilitates ATP production by complex V (ATP synthase). The electron (e^−^) passes through complex I and complex II, respectively, and moves to complex III via the CoQ. Cytochrome c (Cyt c) transfers an electron from complex III to complex IV, which reduces O_2_ to form H_2_O. Electron flow involves proton (H^+^) transfer across the mitochondrial inner membrane at complexes I, III, and IV, creating an electrochemical gradient. Complex V uses the proton motive force to generate ATP.

Mitochondria are organelles with a double-membrane structure containing five complexes in the inner membrane. In complexes I–IV (electron transport chain: ETC), protons are pumped into the intermembrane space during electron transport^4^. The concentration gradient of protons creates a mitochondrial membrane potential (MMP), which is used by complex V (ATP synthase) (Fig. 1). During the process of ATP production, the leaked electrons from the ETC generates ROS^5^, while a reduced form of coenzyme Q (CoQ), known as a representative antioxidant in the ETC, protects against mitochondrial damage caused by ROS as a part of homeostasis mechanism. However, the production of excess ROS over the defense system in problematic situations causes oxidative stress and decreases membrane potential, resulting in an inability to produce energy and mitochondrial dysfunction^6^. Rotenone (ROT), a major complex I inhibitor, interferes with the conversion of CoQ to reduced CoQ (CoQH_2_) by binding to NADH dehydrogenase, which is part of complex I^7,8^. As a result, electrons flow back, and ROS are generated, leading to cell death^9,10^. Therefore, ROT has been widely used in experimental models to explore drug effectiveness against mitochondrial dysfunction^11,12^.

**Figure 2:**
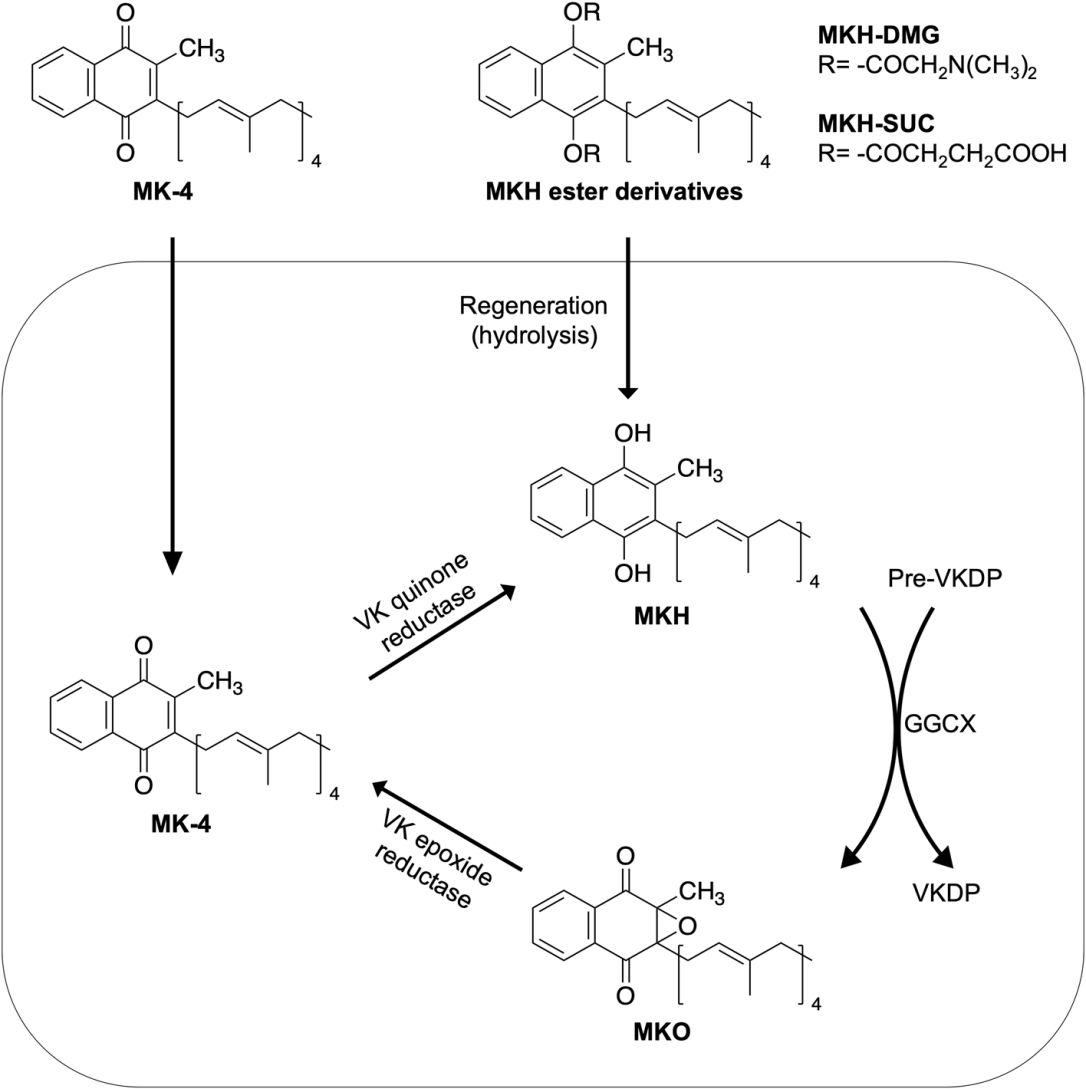
Schematic illustration of the vitamin K cycle and concept of the menahydroquinone-4 delivery system. MKH, menahydroquinone-4; MK-4, menaquinone-4; MKO, menaquinone-4 epoxide; VK, vitamin K; VKDP, vitamin K-dependent protein; GGCX, γ-glutamyl carboxylase.

Menaquinone-4 (MK-4, vitamin K_2(20)_) has been clinically used as a therapeutic agent for osteoporosis in Japan, and its long-term safety has been confirmed^13^. It has been reported that MK-4 suppresses neuronal cell death mediated by ROT-induced microglial activation^14^. However, a recent study showed that vitamin K_2_ is not a substitute for CoQ_10_^15^_._ Other studies have reported that MK-4 can act as a mitochondrial electron transporter^16,17^. Thus, whether MK-4 is effective in mitochondrial dysfunction remains controversial. MK-4 cannot act as a cofactor for γ-glutamyl carboxylase (GGCX) for the post-translational modification of vitamin K-dependent proteins (VKDP) until MK-4 is reduced to menahydroquinone-4 (MKH)^18^. MKH is stoichiometrically converted to MK-4 epoxide (MKO) when it acts as a cofactor for GGCX (Fig. 2). Since MKH has a strong reduction capability, MKH but not MK-4 functions as a ROS scavenger. As a result, it would exert a protective effect against mitochondrial ROS. It has also been revealed that MKH is supplied not only by the reduction of exogenous MK-4 but also by biosynthesizing vitamin K_1_ by UBIAD1^19^ and has been reported to be highly distributed in the brain in the form of MK-4^20^. Therefore, if the efficacy of MK-4 is proven, it is speculated that our study will contribute to the development of safe and effective drugs for intractable diseases such as neurodegenerative disorders. However, MK-4 may not be well delivered to cells^21^.

In our laboratory, we synthesized two MKH ester derivatives, MKH 1,4-bis-*N,N*-dimethylglycinate (MKH-DMG) and MKH 1,4-bis-hemi-succinate (MKH-SUC), which overcome the shortcomings of MK-4. In the present study, we aimed to verify whether MKH delivered by MKH prodrugs affected ROT-induced mitochondrial dysfunction and cell death. We have previously reported that MKH-DMG and MKH-SUC can act as effective MKH prodrugs in vitro and in vivo^21,22^. We have also shown that their MKH delivery via intracellular hydrolysis activation is independent of the reductive activation pathway for MK-4^23,24^. In this study, we evaluated the influence of MKH prodrugs on cell death, decrease in MMP, and intracellular CoQ levels following exposure to mitochondrial inhibitors, mainly ROT, in NIH/3T3 cells.

## Results

### MK-4 and MKH derivatives mitigate ROT-induced cell death

**Figure 3:**
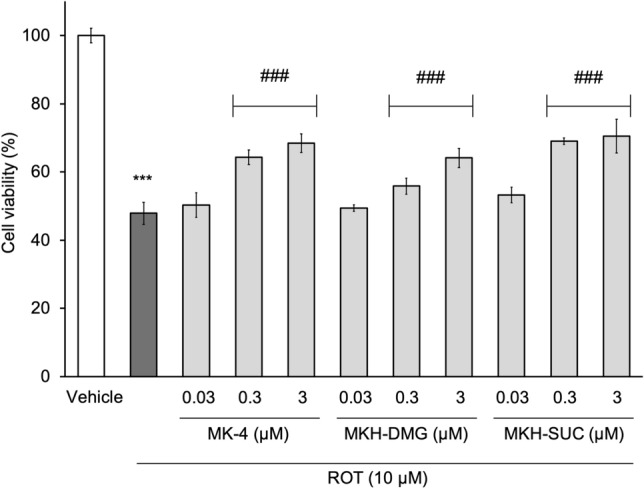
Influence of MK-4 and MKH derivatives on ROT-induced cell death. The NIH/3T3 cells were treated with 0.03‒3 µM MK-4, MKH-DMG, or MKH-SUC for 24 h in the presence of 10 µM ROT. Cell viability was determined using the CTB assay. ***p < 0.001 versus vehicle group (0.1% DMSO + 0.1% ethanol); ^###^p < 0.001 versus ROT only group (Tukey’s test). Mean ± SD (n = 3).

To verify whether MK-4 and MKH derivatives affect ROT (complex I inhibitor)-induced cell death in NIH/3T3 cells, cell viability was assessed using the CellTiter-Blue® (CTB, G8080, Promega Japan, Tokyo, Japan) reagent. As preliminary tests, the cytotoxicity of ROT, MK-4, or MKH derivatives to NIH/3T3 cells was evaluated. ROT treatment (0.01, 0.1, 1, and 10 µM for 24 h) induced a dose-dependent decrease in cell survival rate. The maximum reduction rate in cell viability was approximately 40% of the vehicle group (0.1% dimethylsulfoxide: DMSO) at the highest dose in the 0.01–10 µM dose range (Supplementary Fig. S1). In the absence of ROT, treatment with MK-4 and MKH derivatives for 24 h did not affect cell viability at concentrations of up to 3 µM (Supplementary Fig. S1). Based on the results of the preliminary cytotoxicity tests, the concentrations of the drug treatments were determined as follows: ROT, 10 µM; MK-4 and MKH derivatives, 0.03, 0.3, and 3 µM each. As shown in Fig. 3, the cell viability in the groups treated with MK-4 and MKH derivatives at 0.3 and 3 µM was higher than that with ROT only, while 0.03 µM MK-4 and MKH derivatives did not affect ROT-induced cell death. At 0.3 µM the effects of MK-4 and MKH-SUC treatments reached a plateau, while that of MKH-DMG treatments peaked at 3 μM (Fig. 3). These results showed that the MK-4 and MKH derivatives mitigated ROT-induced cell death.

### MK-4 and MKH derivatives alleviate ROT-induced dysfunction of mitochondria

**Figure 4:**
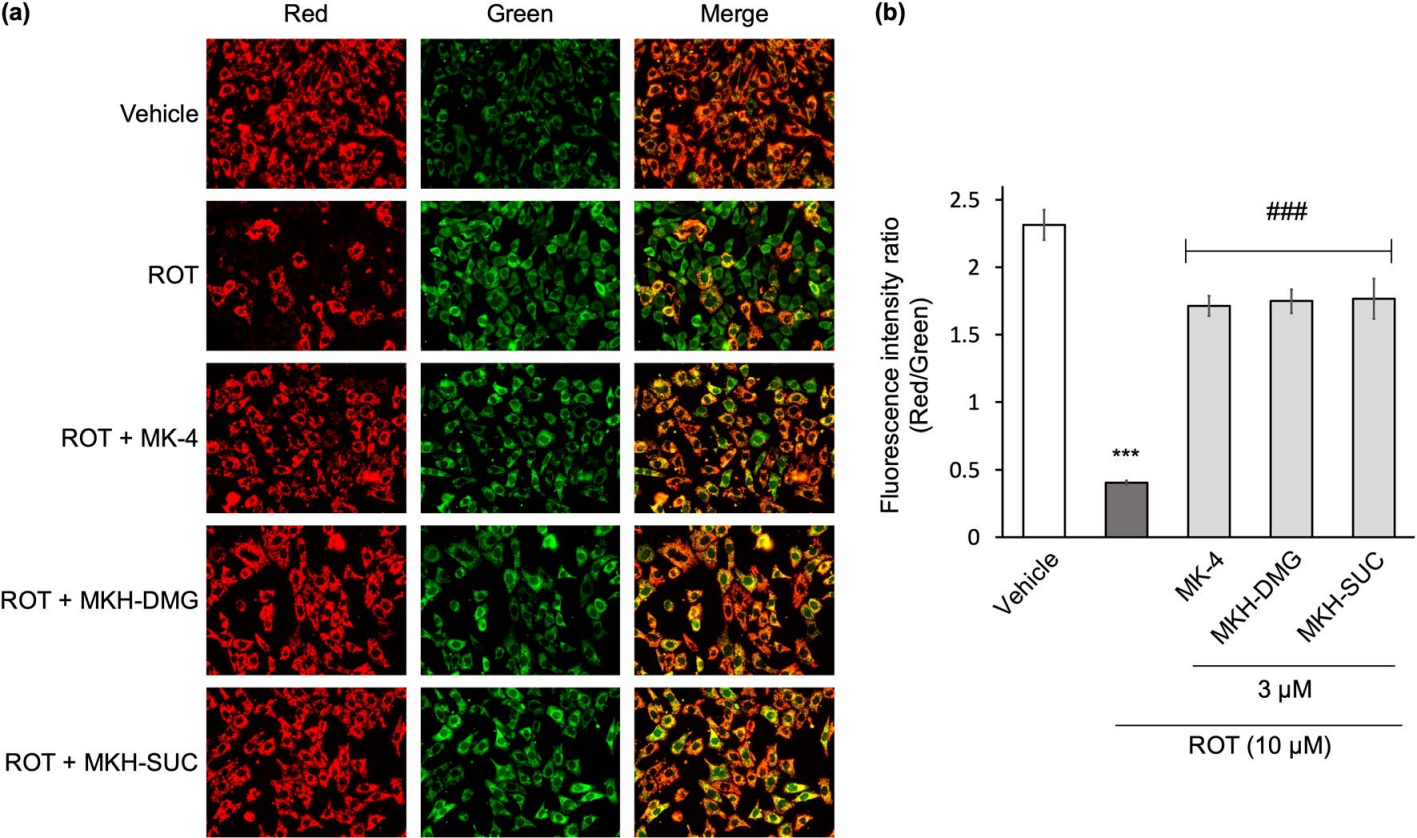
Influence of MK-4 and MKH derivatives on ROT-induced reduction of MMP. The NIH/3T3 cells were treated with 3 µM MK-4, MKH-DMG, or MKH-SUC for 6 h in the presence of 10 µM ROT. MMP was evaluated by JC-1 staining (**a**) Fluorescence microscopic image, (**b**) Fluorescence intensity ratio (Red/Green) quantified using a microplate reader. ***p < 0.001 versus vehicle group; ^###^p < 0.001 ROT only group (Tukey’s test). Mean ± SD (n = 3).

To observe the mitochondrial state before ROT-induced cell death occurs, MMP was evaluated by JC-1 staining. The changes in fluorescence from red to green of JC-1 showed a reduction in MMP. Treatment with 10 µM ROT for 6 h decreased the red fluorescence intensity compared to that in the vehicle group (0.1% DMSO + 0.1% ethanol), as shown in Fig. 4a. The red/green fluorescence intensity ratio was reduced by ROT, as shown in Fig. 4b, indicating mitochondrial dysfunction. Treatment with all three MK-4 and MKH derivatives alleviated the ROT-induced MMP reduction, as shown in Fig. 4a,b. These data indicated that MK-4 and MKH derivatives protected NIH/3T3 cells from ROT-induced mitochondrial damage.

### MK-4 and MKH derivatives suppress ROT-induced ROS

**Figure 5:**
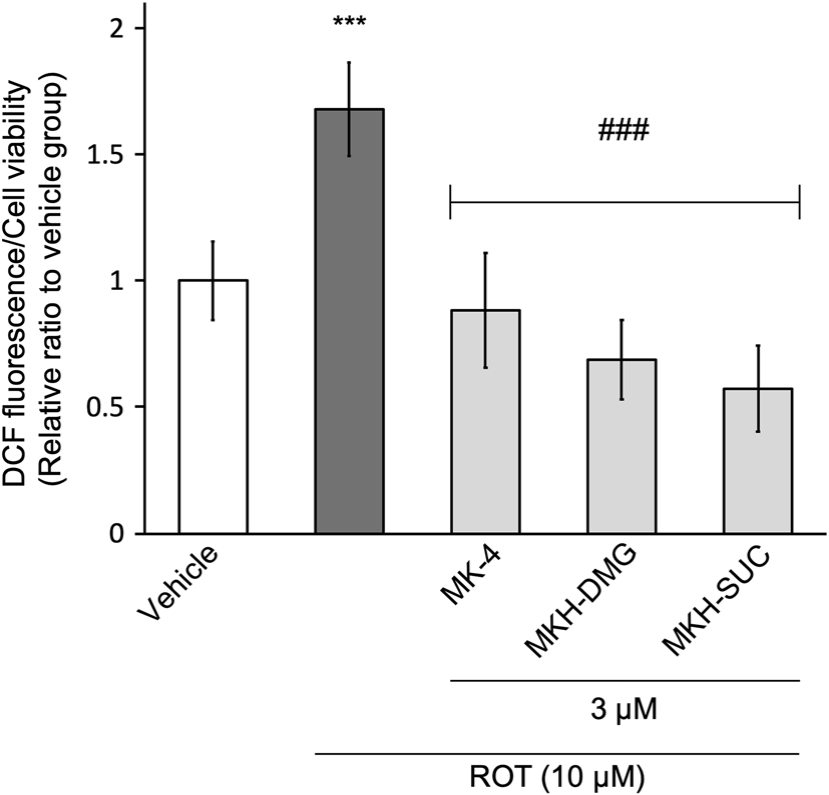
Influence of MK-4 and MKH derivatives on ROT-induced ROS. The NIH/3T3 cells were treated with 3 µM MK-4, MKH-DMG, or MKH-SUC for 6 h in the presence of 10 µM ROT. Intracellular ROS levels were determined by DCFH-DA staining. The relative luminescence unit (RLU) obtained using the DCFH-DA reagent was standardized by RLU from the CTB assay as the living cell number. ***p < 0.001 versus vehicle group; ^###^p < 0.001 versus ROT only group (Tukey’s test). Mean ± SD (n = 3).

ROT is believed to induce ROS in the mitochondria by blocking complex I in the respiratory chain. Therefore, to confirm the influence of MK-4 and MKH derivatives on ROT-induced ROS production, intracellular ROS levels were measured using 2′,7′-dichlorodihydrofluorescein diacetate (DCFH-DA) reagent. The intracellular ROS levels were standardized by the number of living cells, as indicated in Fig. 5. The ROS level in the ROT-only group was 1.7-fold higher than that in the vehicle group. In contrast, the levels of all three MK-4 and MKH derivatives were lower than those of the ROT-only group, indicating similar levels to that of the vehicle group. These data indicated that MK-4 and MKH derivatives maintained intracellular ROS levels in the normal state, even in the presence of ROT.

### MK-4 and MKH derivatives suppress ROT-induced heme oxygenase-1 expression

**Figure 6:**
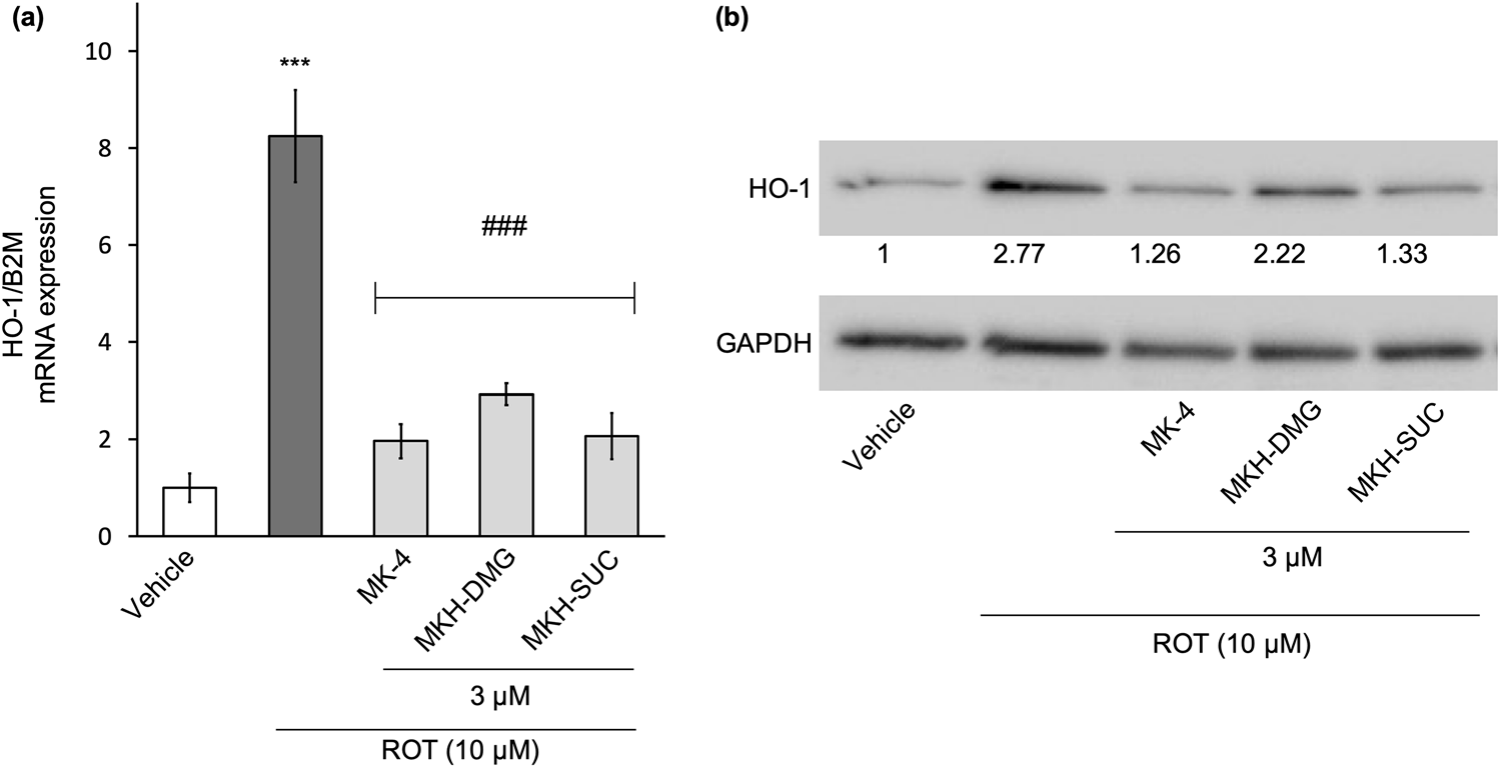
Influence of MK-4 and MKH derivatives on HO-1 induced by ROT. The NIH/3T3 cells were treated with 3 µM MK-4, MKH-DMG, or MKH-SUC for 6 h in the presence of 10 µM ROT. (**a**) HO-1 mRNA level obtained by qPCR. (**b**) HO-1 protein was detected by western blotting. ***p < 0.001 versus vehicle group; ^###^p < 0.001 versus ROT only group (Tukey’s test). Data are presented as mean ± SD (n = 3).

Heme oxygenase-1 (HO-1) is induced by such as ROS and inflammatory cytokines. To evaluate the effect of ROT-induced ROS on the amount of HO-1 expression, real-time PCR and western blotting were performed. HO-1 gene expression increased eightfold in response to ROT compared to vehicle treatment (Fig. 6a). In contrast, the HO-1 gene expression levels of MK-4 and MKH derivatives were 2.7- to 4.0-fold lower than that of ROT (Fig. 6a). HO-1 protein expression was clearly increased by ROT, and the MK-4 and MKH derivatives suppressed ROT-induced HO-1 expression (Fig. 6b). The whole blot image can be found in Supplementary Figure S2. These data show that MK-4 and MKH derivatives suppress ROT-induced HO-1 gene and protein levels, supporting their suppressive effect on ROT-induced ROS.

### MK-4 and MKH derivatives deliver MKH to NIH/3T3 and suppress ROT-induced coenzyme Q_9_ decrease

**Figure 7:**
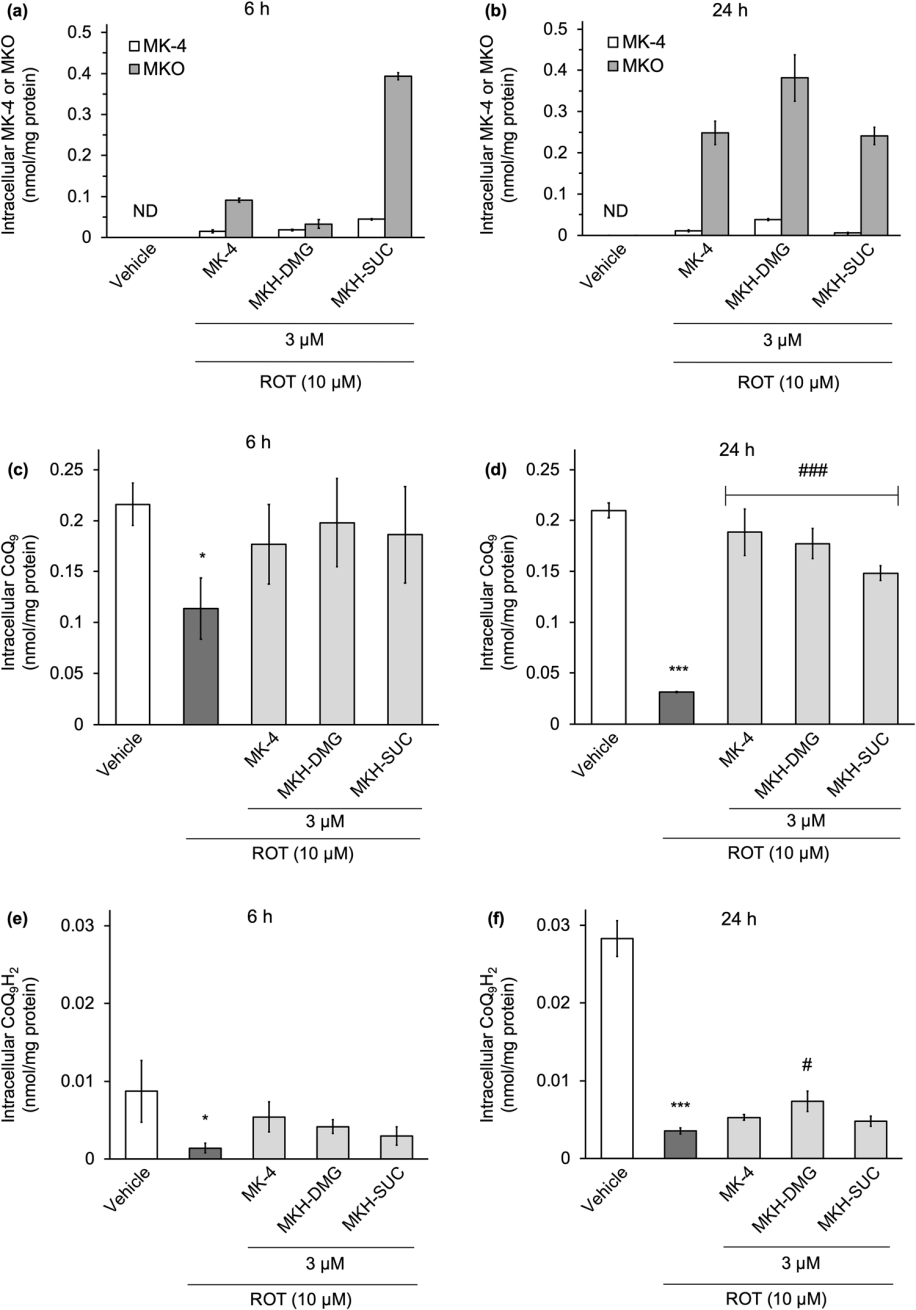
Intracellular MK-4, MKO, and native CoQ_9_/CoQ_9_H_2_ levels in NIH/3T3 cells treated with ROT and MKH derivatives. The NIH/3T3 cells were treated with 3 µM MK-4, MKH-DMG, or MKH-SUC for 6 h or 24 h in the presence of 10 µM ROT. The intracellular levels of MK-4, MKO, CoQ_9_, and CoQ_9_H_2_ were determined by LC–MS/MS. Intracellular MK-4 or MKO levels at (**a**) 6 h and (**b**) 24 h, CoQ_9_ levels at (**c**) 6 h and (**d**) 24 h, CoQ_9_H_2_ levels at (**e**) 6 h and (**f**) 24 h. ***p < 0.001 versus vehicle group; *p < 0.05 versus vehicle group; ^###^p < 0.001 versus ROT only group; #p < 0.05 versus ROT only group (Tukey’s test). Data are presented as mean ± SD (n = 3).

To confirm MKH delivery by MK-4 and MKH derivatives and to investigate the effect of ROT-induced mitochondrial damage on the endogenous coenzyme Q_9_ (CoQ_9_)/reduced coenzyme Q_9_ (CoQ_9_H_2_) balance, we measured intracellular MK-4, MKO, CoQ_9_, and CoQ_9_H_2_ levels using LC–MS/MS. MKH cannot be measured directly because it is easily oxidized to MK-4 in presence of air. The MKO level reflects the amount of MKH delivered due to the conversion of MKH to MKO, which acts as a cofactor for the post-translational modification of VKDP by GGCX. In the vehicle group, neither MK-4 nor MKO was detected. Intracellular MKO was observed in the drug-treated groups, indicating that both the MK-4 and MKH derivatives functioned as MKH prodrugs. After 6 and 24 h of drug treatment, intracellular MKO levels were higher than intracellular MK-4 levels at every time point. The maximum intracellular drug level of MK-4 was approximately 0.05 nmol/mg protein, and that of MKO was approximately 0.4 nmol/mg protein (Fig. 7a,b). These results suggested that the delivered MKH was efficiently utilized in the VKDP production process. The delivery of MKH by MK-4 and MKH derivatives was in the order MKH-SUC > MK-4 > MKH-DMG 6 h after drug treatment (Fig. 7a). After 24 h of treatment, the order of MKH delivery was MKH-DMG > MK-4 ≈ MKH-SUC (Fig. 7b). These results indicated that the ester hydrolysis of MKH-SUC in cells is fast, whereas that of MKH-DMG is steady.

In the CoQ_9_H_2_ measurement, a part of the CoQ_9_H_2_ was oxidized to CoQ_9_ during the extraction process. Although oxidation could not be completely prevented, extraction was carried out promptly and simultaneously for all samples. The intracellular CoQ_9_ level in the ROT-treated group at 6 h decreased compared to that in the vehicle group and reached 10% of the vehicle group after 24 h. Simultaneous treatment with MK-4 or MKH derivatives significantly suppressed the ROT-induced decrease in intracellular CoQ_9_ level, which was about 70‒90% of the vehicle group after 24 h (Fig. 7c,d). On the other hand, the amount of CoQ_9_H_2_ detected was approximately 5‒10% of that of CoQ_9_. ROT treatment markedly reduced CoQ_9_H_2_ after 6 h, but the MK-4 and MKH derivatives slightly affected the low levels of CoQ_9_H_2_ (Fig. 7e,f).

### MK-4 and MKH derivatives protect against 3-NP- and CCCP-induced cell death but not against AA and OA

**Figure 8:**
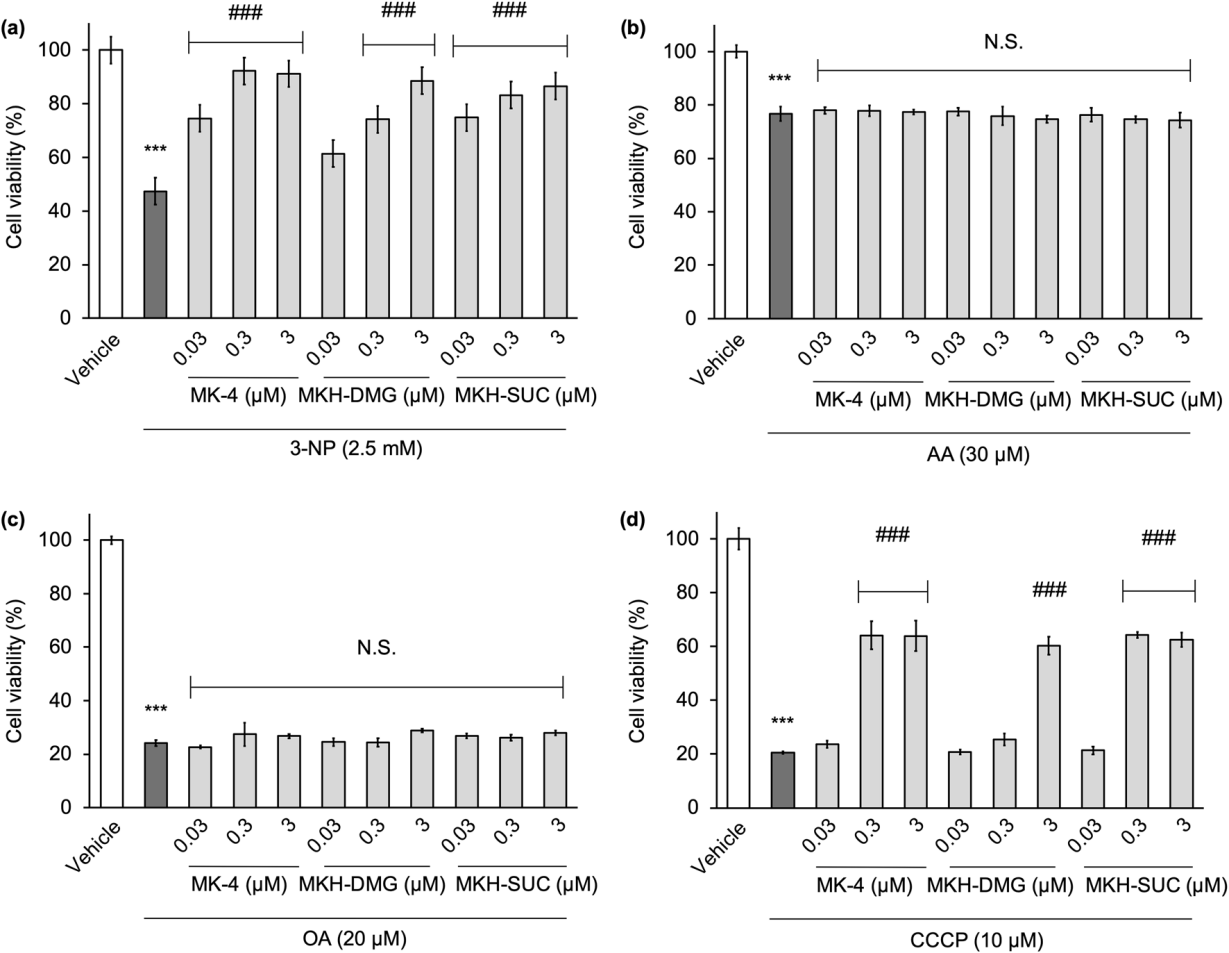
Influence of MK-4 and MKH derivatives on cell death induced by complex-II, III, V, and depolarization mitochondrial inhibitors. The NIH/3T3 cells were treated with 0.03‒3 µM MK-4, MKH-DMG, or MKH-SUC for 24 h in the presence of (**a**) 2.5 mM 3-NP, (**b**) 30 µM AA, (**c**) 20 µM OA, and (**d**) 10 µM CCCP. Cell viability was determined using CTB assay. ***p < 0.001 versus vehicle group; ^###^p < 0.001 versus 3-NP only group or CCCP only group; no significant (N.S.) versus AA only group or OA only group (Tukey’s test). Data are presented as mean ± SD (n = 3).

To elucidate the protective effects of MK-4 and MKH derivatives on other respiratory chain complex inhibitors; 3-nitro propionic acid (3-NP, complex II inhibitor), antimycin A (AA, complex III inhibitor), oligomycin A (OA, complex V inhibitor), and carbonyl cyanide m-chlorophenyl hydrazone (CCCP, uncoupler) were used. Cell viability was measured using CTB reagent, and the concentrations of all inhibitors used in the experiment were determined by preliminary experiments (Supplementary Fig. S3). Each of the inhibitors, 3-NP, AA, OA and CCCP, reduced the cell viability to 50%, 80%, 20%, and 20% of the vehicle group, respectively (0.1% ethanol) (Fig. 8). The MK-4 and MKH derivatives effectively mitigated the cytotoxicity of 3-NP and CCCP in a dose-dependent manner (Fig. 8a,d), whereas they did not affect AA- and OA-induced cell death (Fig. 8b,c).

## Discussion

For vitamin K to exert pharmacological effects in post-translational modification of VKDP, antioxidation, and ROS scavenging, its naphthoquinone scaffold must be converted to naphthohydroquinone (a two-electron reduced form). It has been revealed that MKH (hydroquinone form of MK-4) is supplied not only by the reduction of exogenous MK-4 but also by biosynthesizing from vitamin K_1_ by UBIAD1^19^, and it has been reported that MKH is highly distributed in the brain in the form of MK-4^20^. To test the hypothesis of whether MKH is effective in mitochondrial dysfunction, which may correspond to various pathologies, such as Alzheimer's disease and Parkinson's disease, we investigated the effects of MK-4 and MKH derivatives as prodrugs of MKH on mitochondrial inhibitor-induced cytotoxicity in vitro. Vos et al. reported that MK-4 is responsible for ETC in Drosophila experiments^16^; however, Cerqua et al. showed that MK-4 is not a substitute for CoQ_10_ in mammalian cells^15^. Thus, whether MKH contributes to electron transport remains controversial.

NIH/3T3 cells are derived from mouse fibroblasts and are widely used for assessing basic biological effects such as cytotoxicity and elucidation of molecular mechanisms^25,26^. We employed NIH/3T3 cells to evaluate the essential effects of MKH derivatives on mitochondria. In his study, MK-4 and MKH derivatives showed a partial suppressive effect on NIH/3T3 cell death and mitochondrial dysfunction induced by mitochondrial inhibitors of complex I, II (ROT, 3-NP) and uncoupler (CCCP). Moreover, MK-4 and MKH derivatives acted as MKH prodrugs in NIH/3T3 cells and suppressed excessive ROS production and loss of endogenous CoQ_9_ induced by ROT. Based on these results, it is speculated that MKH is a key player in mitigating mitochondrial dysfunction induced by mitochondrial inhibitors.

The effect and delivery of MKH-DMG were slower and milder than those of MK-4 and MKH-SUC (Figs. 3, 7a,b). Our previously published reports show a correlation between intracellular MKH delivery and efficacy, consistent with our results^23,24^. If MKH delivery is important for its suppressive effect on mitochondrial dysfunction, it can be expected that the efficacy of MKH-DMG at low therapeutic concentrations will be lower than those of MK-4 and MKH-SUC. Therefore, it seems reasonable that the suppressive effects of MKH-DMG on the increase in cell death induced by mitochondrial inhibitors were lower than those of MK-4 and MKH-SUC (Figs. 3, 8a,d).

ROT treatment induced a significant decrease in MMP, increased ROS production, and increased HO-1 expression at early time points (Figs. 4, 5, 6). Xiong et al. showed that MMP reduction and ROS production occurred at an early stage after drug treatment, with ROS production occurring earlier^27^. Furthermore, Iacobini et al. suggest that ROT-induced ROS generation and MMP reduction are early events in cell death^28,29^, and Geng et al. showed that short-term ROT treatment affected ROS production and MMP^30^. Therefore, our results indicated that the production of ROS induced a decrease in MMP, resulting in cell death. HO-1 is a stress response protein induced by oxidative stress, heavy metals, and cytokines, and exhibits cytoprotective and antioxidant activities^31,32^. ROS are representative factors that induce oxidative stress, and inhibition of ROS accumulation reduces HO-1 expression^33^. Thus, the level of HO-1 expression reflects the amount of ROS production. Our results showed that ROT exposes cells to stress (Fig. 6), and we speculate that it is because of ROS. MK-4 and MKH derivatives significantly suppressed the decrease in MMP, increase in ROS production and increase in HO-1 expression (Figs. 4, 5, 6). Therefore, we hypothesize that MK-4 and MKH derivatives relieved stress on cells by suppressing ROS overproduction, resulting in reduced MMP depletion and cell death.

CoQ_9_ (oxidized form) and CoQ_9_H_2_ (two-electron reduced form) are electron transporters in the mitochondria, and CoQ_9_ is more abundant than CoQ_10_ in rodents. NADH dehydrogenase in complex I activate the ETC by reducing CoQ_9_–CoQ_9_H_2_. The MK-4 and MKH derivatives maintained endogenous CoQ_9_ even in the presence of ROT (Fig. 7c,d). The ratio of CoQ_9_H_2_/CoQ_9_ in NIH/3T3 cells in the vehicle group was low (Fig. 7c,e). To our knowledge, although no data indicating the ratio in NIH/3T3 cells have been found, low ratios have been observed in certain tissues, excluding the liver, adipose, and plasma^34,35^. Previous reports have demonstrated that ROT, an NADH dehydrogenase inhibitor, increases the ratio of CoQ_9_/CoQ_9_H_2_ within a short period of drug treatment^36,37^. In the present study, ROT treatment decreased not only CoQ_9_H_2_ (Fig. 7e,f) but also CoQ_9_, particularly at 24 h (Fig. 7d), suggesting that the absolute amount of CoQ_9_ was lost due to ROT-induced mitochondrial collapse. Remarkably, CoQ_9_H_2_ remained low despite the high CoQ_9_ levels maintained by MK-4 and MKH derivatives (Fig. 7d,f). This result suggests that ROT strongly blocks the reduction of CoQ_9_ to CoQ_9_H_2_ independent of normal CoQ_9_ levels. In other words, MKH prodrugs may effectively suppress ROT-induced ROS production via (i) ROS quenching or (ii) electron transport in complex I instead of CoQ. Menke et al. reported the protective effect of CoQ_10_ on ROT-induced apoptosis in SH-SY5Y neuroblast cells, suggesting that excessive exogenous CoQ_10_ may overcome ROT inhibition via a bypass mechanism for the ETC^38^.

It has been reported that the main sites of ROS generation in mitochondria are in complex I and III^39–41^, particularly in complex I^42^. AA inhibits the reduction of CoQ in respiratory chain complex III and induces ROS production^43^. However, it has also been reported that ROS are generated not only in ETCs I and III but also in ETCs II and IV^44^, 3-NP, which irreversibly inhibits succinate dehydrogenase, generates ROS and promotes mitochondrial damage^45^. Furthermore, studies with CCCP, which reduces MMP and inhibits oxidative phosphorylation, and OA, which binds to the proton pump of complex V and inhibits ATP synthesis, confirm ROS generation^46,47^. Regarding the inhibitory effect of MK-4 and MKH derivatives on cell death induced by 3-NP and CCCP, it is necessary to further investigate whether the effects of MKH prodrug are due to the normalization of ROS levels and ROT. However, it is unclear why MK-4 and MKH derivatives have no effect on OA- and AA-induced cell death. Although Cerqua et al. showed that MK-4 does not function as a substitute for CoQ by using CoQ-depleted cells, the ROT used in our study did not block CoQ biosynthesis and did not lead to CoQ depletion in the mitochondria^15^. Therefore, MKH may be able to assist or substitute for CoQ to drive the ETC. Another possibility is that the increased MKO levels by MKH prodrugs reflect the production of VKDP, and induced VKDP may affect mitochondrial rescue. Further clarification of the mechanisms underlying the suppressive effects of MKH prodrugs on mitochondrial dysfunction, including the mechanism by which MKH prodrugs maintain ROS at normal levels, is required.

In this study, we present the basic effects of MKH derivatives on mitochondrial dysfunction and confirmed reproducibility. MK-4 and MKH derivatives have a partial inhibitory effect on cell death induced by respiratory chain complex I and II inhibitors and uncouplers and suppress ROS generation in mitochondria. These compounds functioned as prodrugs of MKH in NIH/3T3 cells, and it was considered that MKH delivery exerts a suppressive effect on cell death and ROS production. It is believed that the evaluation of the effect of MKH prodrugs on mitochondrial dysfunction and elucidation of the detailed mechanism using NIH/3T3 cells will become a foundation for further developmental research using cells derived from various organs.

## Materials and methods

### Chemicals

Menaquinone-4 (MK-4) was purchased from Seebio Biotech Inc. (Shanghai, China). Menahydroquinone-4 1,4-bis-*N,N*-dimethylglycinate hydrochloride (MKH-DMG), and menahydroquinone-4 1,4-bis-hemi-succinate (MKH-SUC) were synthesized in our laboratory using previously reported methods^48^. The following chemicals were obtained commercially: rotenone (R8875), 3-nitropropionic acid (N5636), and antimycin A from *Streptomyces* sp. (A8674) from Sigma-Aldrich (St. Louis, MO, USA); oligomycin A (AG-CN2-0517) from AdipoGen Life Sciences, Inc. (San Diego, CA, US); carbonyl cyanide m-chlorophenylhydrazone (CCCP; 034-16993) from FUJIFILM Wako Pure Chemical Corporation (Osaka, Japan); and coenzyme Q_9_ (CoQ_9_) from Cayman Chemical (Ann Arbor, MI, USA). The following antibodies were used: heme oxygenase 1 (HO-1) from Cell Signaling Technology (Danvers, MA, USA) and monoclonal mouse anti-glyceraldehyde-3-phosphate dehydrogenase (GAPDH) from Sigma-Aldrich.

### Cell culture

The mouse fibroblast-like cell line NIH/3T3 (RCB2767) was obtained from the RIKEN BioResource Research Center (Ibaraki, Japan). Cells were maintained in Dulbecco's Modified Eagle Medium/Nutrient Mixture F-12 (DMEM/F-12; Thermo Fisher Scientific, Waltham, MA, USA) containing 10% fetal bovine serum (FBS; HyClone Standard Fetal Bovine Serum Collected and Processed in USA, Cytiva, Tokyo, Japan) and 1% penicillin/streptomycin (Thermo Fisher Scientific) at 37 °C under humidified 5% CO_2_ atmosphere.

### Cell viability assay

Cell viability was assessed using the CellTiter-Blue® (CTB, Promega) by the following cell viability assay method. NIH/3T3 cells were plated at a density of 1.0 × 10^4^ cells per well in 96-well black plates and allowed to attach for 24 h. Then, drug media, including 10 µM of ROT and 0.03‒3 µM of MK-4, MKH-DMG, or MKH-SUC at the different concentrations indicated in the related result section, were exposed for 24 h. Fluorescence signals as cell viability were measured using a microplate reader (Infinite M200 PRO, Tecan, Kanagawa, Japan) according to the manufacturer’s instructions.

### Measurement of the mitochondrial membrane potential

MMP in cells was assessed using the JC-1 MitoMP Detection Kit (Dojindo Lab, Kumamoto, Japan) according to the manufacturer’s instructions. JC-1 accumulated in mitochondria forms red fluorescent aggregates at high membrane potentials, whereas JC-1 monomers show green fluorescence, indicating low membrane potentials. For the analysis of MMP, NIH/3T3 cells were cultured in a collagen-I-coated 96-well black plate at a density of 1.0 × 10^4^ cells/well or in a collagen-I-coated glass dish (IWAKI, AGC TECHNO GLASS Co. Ltd, Shizuoka, Japan) at a density of 2.5 × 10^5^ cells/dish. The cells were allowed to adhere for 24 h. The cells were then treated with MK-4, MKH-DMG, or MKH-SUC in the presence of ROT for 6 h. Then, the cells were stained with JC-1 (1 mg/mL in DMSO) and incubated at 37 °C for 30 min in the dark. The cells were washed twice with serum-free media, visualized under a fluorescence microscope (BZ-X810, KEYENCE, Osaka, Japan), and quantified using a microplate reader (Infinite M200 PRO) (red; Ex/Em = 561/595 nm, green; Ex/Em = 488/540 nm).

### Intracellular ROS measurement

Intracellular ROS levels were examined using DCFH-DA (Thermo Fisher Scientific). The DCFH-DA probe is rapidly oxidized by ROS and converted into fluorescent 2′,7′-dichlorodihydrofluorescein (DCF). The DCF fluorescence intensity is proportional to the ROS level in the cytoplasm. Briefly, NIH/3T3 cells were seeded at a density of 1.0 × 10^4^ cells/well in a collagen-I-coated 96-well black plate and allowed to adhere for 24 h. The cells were treated with MK-4, MKH-DMG, or MKH-SUC in the presence of ROT for 6 h. The cells were washed once with serum-free media and incubated in 100 µL of 10 µM DCFH-DA for 30 min in the dark at 37 °C. After incubation, intracellular fluorescence was measured at excitation and emission wavelengths of 485 and 530 nm, respectively, using a microplate reader (Infinite M200 PRO). The obtained ROS levels were standardized by cell viability using the CTB reagent, as described in the Cell Viability Assay section.

### Real-time quantitative PCR

Total RNA was extracted from cultured cells using a High Pure RNA Isolation Kit (Roche Diagnostics K.K., Tokyo, Japan). cDNA was reverse-transcribed using the ReverTra Ace-a-reverse transcription kit (Toyobo, Shanghai, China), and quantification was performed using LightCycler® 480 SYBR Green I Master (Roche Diagnostics K.K.). The sequences of the primers used were as follows: heme oxygenase-1 (HO-1), forward primer: 5′-CACTCTGGAGATGACACCTGAG-3′; reverse primer: 5′- GTGTTCCTCTGTCAGCATCACC-3′; beta-2 microglobulin (B2M), forward primer: 5′- ACGTAGCAGTTCAGTATGTTCG-3′; reverse primer: 5′- GGTCTTTCTGGTGCTTGTCT-3′. B2M was used as an internal control.

### Western blot analysis

NIH/3T3 cells were seeded at a density of 4.0 × 10^5^ cells/dish in a collagen-I coated 60 mm dish and treated with 10 µM ROT with or without 3 µM MK-4, MKH-DMG, or MKH-SUC for 6 h. Cultured cells were washed with ice-cold PBS and lysed in RIPA buffer (0.5% NP-40, 0.25% sodium deoxycholate, 0.05% SDS, 150 mM NaCl, and 50 mM HEPES, pH 7.4) containing a protease inhibitor cocktail (Nacalai Tesque, Kyoto, Japan) on ice. A plastic cell scraper was used to scrape the adherent cells. Cell lysates were then clarified by centrifugation at 20,600 × *g* at 4 °C for 15 min, and the supernatant was collected. The total protein concentration was determined using the Pierce™ BCA protein assay kit (Thermo Fisher Scientific). Proteins (every 10 μg) were separated by SDS-PAGE using Super Sep ™ Ace 15% 13-well gels (FUJIFILM Wako) and transferred onto PVDF membranes (Bio-Rad, Hercules, CA, USA). The membrane was blocked using Blocking One solution (Nacalai Tesque) and incubated with anti-HO-1 (1:1,000) and anti-GAPDH antibodies (1:10,000), respectively, at 25 °C for 1 h. After washing, the membranes were treated with the appropriate secondary antibodies and visualized using Immunostar LD (FUJIFILM Wako). Protein expression was analyzed using the ImageJ software (version 1.53 k).

### Determination of intracellular MKO, MK-4, CoQ_9_, and CoQ_9_H_2_

NIH/3T3 cells were seeded at 3.0 × 10^5^ cells/well in 6-well plates and allowed to attach for 24 h. Next, the cells were cultured in a medium with or without 3 µM MK-4, MKH-DMG, or MKH-SUC in the presence of 10 µM ROT. The medium was then removed, and the cells were washed twice with PBS. The cells were collected in 1 mL PBS and sonicated on ice. Cell homogenates were combined with an equal volume of ethanol and three times the volume of n-hexane, vortexed for 2 min, and centrifuged at 1750 × *g* for 10 min. The organic layer was evaporated using N_2_ gas. The residue was reconstituted with 200 µL of ethanol and analyzed using LC–MS/MS, as described below. The protein concentration in the cell homogenate was determined using a BCA protein assay kit (Thermo Fisher Scientific).

### LC–MS/MS

LC–MS/MS was performed using an LC–MS-8060 liquid chromatography-mass spectrometer (Shimadzu, Kyoto, Japan) and a Shimadzu UFLC System (Shimadzu, Kyoto, Japan). Separations were performed on a Shim-pack XR-C8 column (φ 2.2 µm, 3 × 75 mm, Shimadzu) using a mobile phase comprising 10 mM ammonium acetate and 0.1% acetic acid in methanol (pump A) and ethanol (pump B) under gradient elution, at a flow rate of 0.2 mL/min. A binary gradient was established as follows: (A) 10 mM ammonium acetate and 0.1% acetic acid in methanol and (B) 10 mM ammonium acetate and 0.1% acetic acid in ethanol; 30% B at 0 min, 30% B at 2.00 min, 70% B at 10.00 min, 70% B at 10.01 min, and 70% B at 12.00 min. The column temperature was maintained at 40 °C. The mass spectrometer was equipped with an electrospray ionizer and operated in the positive ion mode. Identification and quantitation were performed under the MS/MS-multiple reaction monitoring (MRM) mode, using the following transition ions: m/z 461.0 → 81.0 for the [M + H]^+^ MKO adduct; m/z 445.0 → 187.0, for the [M + H]^+^ MK-4 adduct; m/z 815.2 → 197.0, for the [M + NH_4_]^+^ CoQ_9_H_2_ adduct; and m/z 796.2 → 197.0, for the [M + H]^+^ CoQ_9_ adduct. Retention times were as follows: MKO, 3.5 min; MK-4, 3.9 min; CoQ_9_H_2_, 5.5 min; and CoQ_9_, 7.4 min.

### Statistical analysis

Comparisons among groups were performed by one-way ANOVA with Tukey’s test, and the analyses were carried out using GraphPad Prism 6 (GraphPad Software, San Diego, CA, USA); results with p < 0.05 were considered significant.

## Supplementary Information

Supplementary Information.

## Data Availability

The datasets generated during and/or analyzed during the current study are available from the corresponding author upon reasonable request.
