# Supplementary material for: Delivery of the reduced form of vitamin K2(20) to NIH/3T3 cells partially protects against rotenone induced cell death
Source: Sci Rep. 2022 Nov 18;12:19878. doi: 10.1038/s41598-022-24456-3 (PMC9674836; doi:10.1038/s41598-022-24456-3)
Supplement: Supplementary file 1 — Supplementary Information. [file 41598_2022_24456_MOESM1_ESM.pdf]

Delivery of the reduced form of vitamin $K_{2(20)}$ to NIH/3T3 cells partially protects against rotenone induced cell death

Erina Toki[1], Shotaro Goto[1], Shuichi Setoguchi[1], Kazuki Terada[2], Daisuke Watase[1], Hirofumi Yamakawa[3], Ayano Yamada[1], Mitsuhisa Koga[1], Kaori Kubota[1], Katsunori Iwasaki[1], Yoshiharu Karube[1], Kazuhisa Matsunaga[1]* and Jiro Takata[1]

[1]Faculty of Pharmaceutical Sciences, Fukuoka University, Fukuoka 814-0180, Japan
[2]Faculty of Pharmaceutical Sciences, Himeji Dokkyo University, Himeji 670-8524, Japan
[3]Radioisotope Center, Fukuoka University, Fukuoka 814-0180, Japan
*k-matsu@fukuoka-u.ac.jp

Supplementary Figures

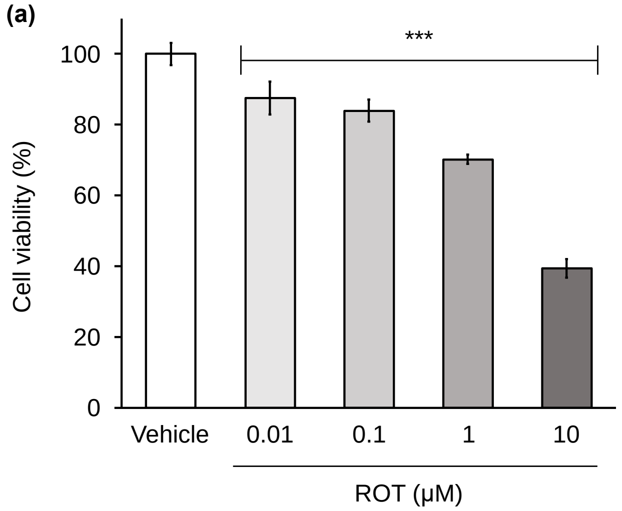

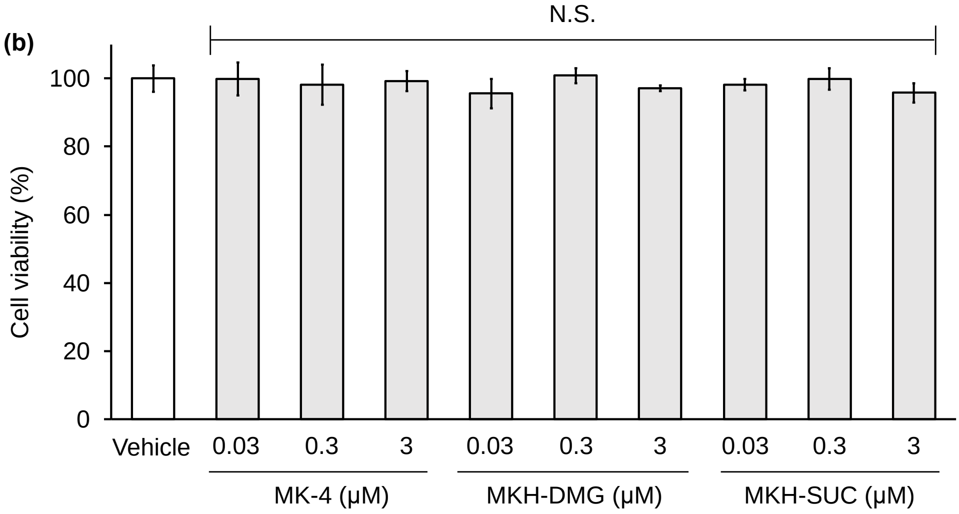

Supplementary Figure S1. Effect of ROT, MK-4, and MKH derivatives on NIH/3T3 cell viability

NIH/3T3 cells were treated with 0.01–10 μM ROT (**a**), 0.03–3 μM MK-4, MKH-DMG, or MKH-SUC (**b**) for 24 h. Cell viability was determined by the CellTiter-Blue® (CTB, G8080, Promega Japan, Tokyo, Japan) assay. ***, $p < 0.001$ vs. vehicle group (Tukey's test). Data are presented as mean ± SD (n=3).

(a) (b)

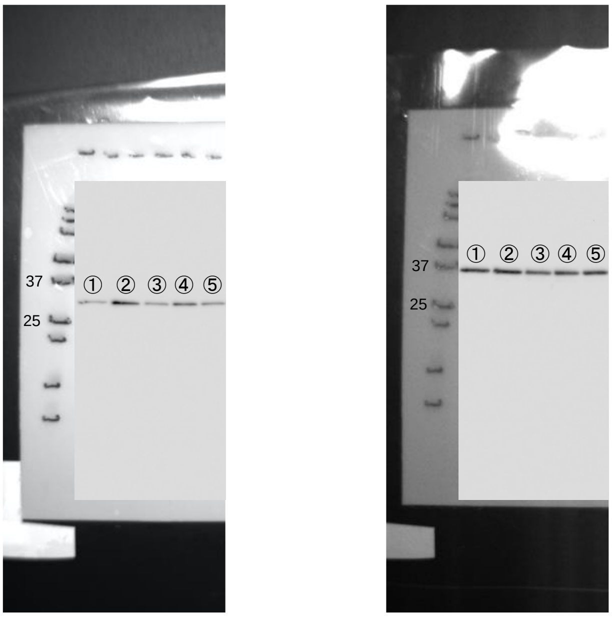

HO-1 (28 kDa) 1:1000 GAPDH (37 kDa) 1:10000

① vehicle
② ROT (10μM)
③ ROT (10μM) + MK-4 (3μM)
④ ROT (10μM) + MKH-DMG (3μM)
⑤ ROT (10μM) + MKH-SUC (3μM)

Supplementary Figure S2. The unprocessed western blots images.

The NIH/3T3 cells were treated with 3 μM MK-4, MKH-DMG, or MKH-SUC for 6 h in the presence of 10 μM ROT. (a) The bright field image of the HO-1 treated membrane and the unprocessed band for the HO-1 protein; (b) the bright field image of the GAPDH-treated membrane and the unprocessed band of the GAPDH protein.

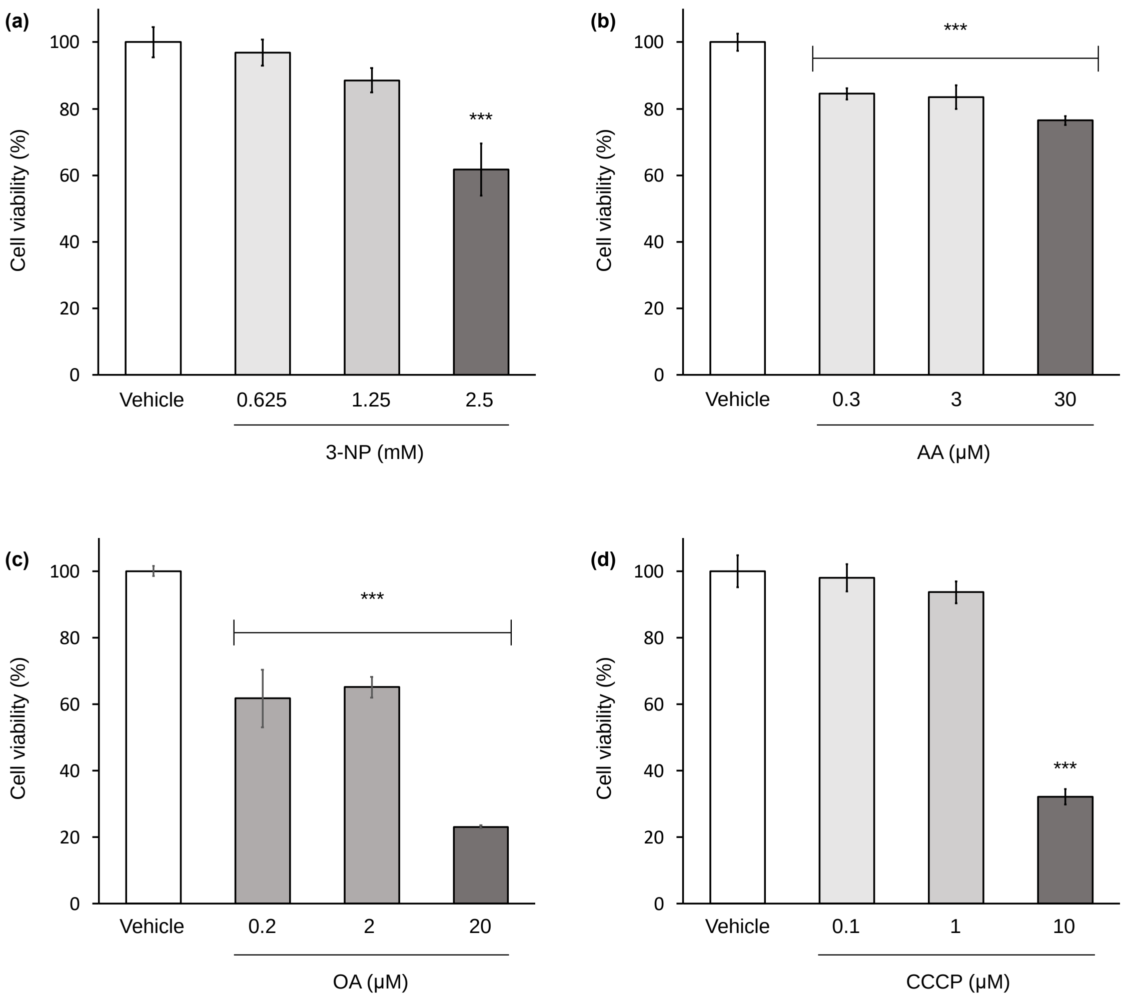

Supplementary Figure S3. Effect of mitochondrial inhibitors and mitochondrial uncouplers on NIH/3T3 cell viability

The CTB assay was performed to assess the cell viability after drug treatment for 24 h. NIH/3T3 cells were treated with 0.625–2.5 mM 3-NP (**a**), 0.3–30 μM AA (**b**), 0.2–20 μM OA (**c**), 0.1–10 μM CCCP (**d**). ***, $p < 0.001$ vs. vehicle group (Tukey's test). Data are presented as mean ± SD (n=3).
